# 

**Published:** 1868-02-15

**Authors:** 


### CHICAGO MEDICAL JOURNAL.

Edited by J. ADAMS ALLEN, M.D., LL.D.,

Professor of Principles and Practice of Medicine and Clinical Medicine in Rush Medical College.

All letters for the JOURNAL should be addressed to Dr. J. ADAMS ALLEN, P. O. Box 1948, Chicago, Ill.

TERMS — $3 Per Annum, in Advance.

$4 if not paid before 1st of July, in each year. Single copies, 25 cents.

### *Spring Course of Instruction in Rush Medical College.*

This course, it will be remembered, opens on the first Monday of March ensuing, and continues during three months. Daily lectures, recitations, and clinics at the college and hospitals of the city. Free to all matriculants of the college and graduates in medicine. For details address the Secretary of the College, Prof. DeLaskie Miller, M.D., 518 Wabash Avenue, Chicago.

There will be daily clinics at the college throughout the entire year. In fact, opportunities for medical students, the present year in this city are largely superior to any which have heretofore existed, and, it is fair to say, fully equal their wants.

